# A Malaria Parasite Cross Reveals Genetic Determinants of *Plasmodium falciparum* Growth in Different Culture Media

**DOI:** 10.3389/fcimb.2022.878496

**Published:** 2022-05-30

**Authors:** Sudhir Kumar, Xue Li, Marina McDew-White, Ann Reyes, Elizabeth Delgado, Abeer Sayeed, Meseret T. Haile, Biley A. Abatiyow, Spencer Y. Kennedy, Nelly Camargo, Lisa A. Checkley, Katelyn V. Brenneman, Katrina A. Button-Simons, Manoj T. Duraisingh, Ian H. Cheeseman, Stefan H. I. Kappe, François Nosten, Michael T. Ferdig, Ashley M. Vaughan, Tim J. C. Anderson

**Affiliations:** ^1^Center for Global Infectious Disease Research, Seattle Children’s Research Institute, Seattle, WA, United States; ^2^Program in Disease Intervention and Prevention, Texas Biomedical Research Institute, San Antonio, TX, United States; ^3^Department of Biological Sciences, Eck Institute for Global Health, University of Notre Dame, Notre Dame, IN, United States; ^4^Immunology and Infectious Diseases Department, Harvard T.H. Chan School of Public Health, Boston, MA, United States; ^5^Program in Host Pathogen Interactions, Texas Biomedical Research Institute, San Antonio, TX, United States; ^6^Department of Pediatrics, University of Washington, Seattle, WA, United States; ^7^Shoklo Malaria Research Unit, Mahidol-Oxford Tropical Medicine Research Unit, Faculty of Tropical Medicine, Mahidol University, Mae Sot, Thailand; ^8^Centre for Tropical Medicine and Global Health, Nuffield Department of Medicine Research building, University of Oxford, Oxford, United Kingdom

**Keywords:** bulk segregant analysis, genetic cross, serum, AlbuMAX, *Plasmodium falciparum*

## Abstract

What genes determine *in vitro* growth and nutrient utilization in asexual blood-stage malaria parasites? Competition experiments between NF54, clone 3D7, a lab-adapted African parasite, and a recently isolated Asian parasite (NHP4026) reveal contrasting outcomes in different media: 3D7 outcompetes NHP4026 in media containing human serum, while NHP4026 outcompetes 3D7 in media containing AlbuMAX, a commercial lipid-rich bovine serum formulation. To determine the basis for this polymorphism, we conducted parasite genetic crosses using humanized mice and compared genome-wide allele frequency changes in three independent progeny populations cultured in media containing human serum or AlbuMAX. This bulk segregant analysis detected three quantitative trait loci (QTL) regions [on chromosome (chr) 2 containing aspartate transaminase *AST*; chr 13 containing *EBA-140;* and chr 14 containing cysteine protease *ATG4*] linked with differential growth in serum or AlbuMAX in each of the three independent progeny pools. Selection driving differential growth was strong (*s* = 0.10 – 0.23 per 48-hour lifecycle). We conducted validation experiments for the strongest QTL on chr 13: competition experiments between ΔEBA-140 and 3D7 wildtype parasites showed fitness reversals in the two medium types as seen in the parental parasites, validating this locus as the causative gene. These results (i) demonstrate the effectiveness of bulk segregant analysis for dissecting fitness traits in *P. falciparum* genetic crosses, and (ii) reveal intimate links between red blood cell invasion and nutrient composition of growth media. Use of parasite crosses combined with bulk segregant analysis will allow systematic dissection of key nutrient acquisition/metabolism and red blood cell invasion pathways in *P. falciparum.*

## Introduction

Asexual blood stage malaria parasite growth rates are determined by factors including efficiency of red blood cell (RBC) invasion, nutrient acquisition and proliferation within red blood cells. Not surprisingly, genes underlying these processes are promising targets for vaccine and antimalarial development. For example, parasite genes underlying RBC invasion, such as apical membrane antigen (*AMA1*), are the focus of vaccine development efforts (Draper et al., 2015). Among existing drugs, artemisinin (ART) - the frontline drug against malaria - is activated by hemoglobin digestion (Wang et al., 2015; Birnbaum et al., 2020; Yang et al., 2020), while chloroquine (CQ) interferes with heme polymerization into non-toxic hemozoin (Shafik et al., 2020), and antifolate drugs (pyrimethamine and sulfadoxine) are competitive inhibitors that interrupt the folate biosynthesis pathway (Gregson and Plowe, 2005). The mutations conferring resistance to these drugs are also involved with parasite nutrition transport/metabolism pathways. For example, resistance to ART, is mediated by mutations in *kelch13*, which is required for hemoglobin endocytosis (Birnbaum et al., 2020). Mutations in the CQ resistance transporter (*PfCRT*), which normally functions as a transport channel for ions and peptides mediate resistance to a variety of drugs including CQ and piperaquine (Shafik et al., 2020). Mutations in dihydrofolate reductase and dihydropteroate synthase, components of the folate synthesis pathway confer resistance to pyrimethamine and sulfadoxine (Gregson and Plowe, 2005). Given the importance of RBC invasion and nutrient acquisition/metabolism, effective methods for locating genes and pathways involved in these processes are urgently needed.

Exploiting natural variation in parasite nutrient acquisition and metabolism pathways provides one promising approach. Nguitragool *et al.* used a human malaria parasite *Plasmodium falciparum* genetic cross conducted in chimpanzee hosts to identify an important channel (plasmodial surface anion channel, PSAC) involved in ion transport (Nguitragool et al., 2011). Similarly, Wang *et al.* used a comparable linkage mapping approach to investigate the ability of parasites to utilize exogenous folate (Wang et al., 1999), as this is important for determining the success of drugs that target the parasite folate synthesis pathway. These linkage analysis studies demonstrate how differences in metabolism or nutrient acquisition between parasites can be effectively exploited to better understand the genetic underpinning of metabolic pathways and transport systems, which in turn can highlight potential targets for intervention. However, traditional linkage mapping, requiring phenotyping and sequencing of individual cloned progeny, is laborious and does not access the full power of independent recombinants available in uncloned populations.

Our central aim was to evaluate the efficacy of genetic crosses and a rapid linkage mapping method - bulk segregant analysis - for understanding the genetic basis of nutrition-related phenotypes in *P. falciparum* using differential growth and fitness in serum- or AlbuMAX-based *in vitro* culture as a test system. Bulk segregant analysis provides a simple and fast approach to identify loci that contribute to complex traits (Ehrenreich et al., 2010) that are typically identified using traditional QTL mapping. Using sequencing of pooled progeny populations, bulk segregant analysis measures changes in allele frequency following distinct selections (here comparing asexual blood stage growth in serum or AlbuMAX). Bulk segregant analysis, also referred to as linkage group selection (LGS) (Culleton et al., 2005), has been extensively used with genetic crosses of rodent malaria parasites to map genes determining blood stage multiplication rate, virulence and immunity in *Plasmodium yoelii* (Pattaradilokrat et al., 2009; Abkallo et al., 2017), as well as mutations conferring ART resistance and strain-specific immunity in *Plasmodium chabaudi* (Hunt et al., 2010). Here we use the term bulk segregant analysis in preference to LGS since bulk segregant analysis has been widely used across multiple fields of biology since 1991 (Michelmore et al., 1991), while LGS was an analogous term independently coined by researchers working on rodent malaria parasites (Culleton et al., 2005). As such bulk segregant analysis is more broadly understood across multiple fields. Carrying out genetic crosses in *Anopheles* mosquitoes and human hepatocyte-chimeric mice (FRG huHep mice) (Vaughan et al., 2015) now allows us to routinely generate large pools of recombinant *P. falciparum* progeny without the need for chimpanzee hosts. We have previously generated four independent *P. falciparum* genetic crosses with large numbers of unique recombinant progeny using this approach (Button-Simons et al., 2021), reviewed in (Vendrely et al., 2020).

Continuous *in vitro* culture of asexual blood stages of the malaria parasite *P. falciparum* typically requires human RBCs, buffered RPMI 1640 medium and human serum, with a low oxygen atmosphere at 37°C (Trager and Jensen, 1976). RPMI 1640 medium is the main resource for sugar (glucose), salts, essential amino acids and multiple vitamins (Moore et al., 1967). Human hemoglobin can supply amino acids other than isoleucine (Sherman, 1977), while human serum provides most of the other nutrients needed for parasite growth, such as inorganic and organic cations. Lipid-enriched bovine albumin (AlbuMAX) is the most widely used human serum substitute and extensively used for blood stage parasite culture. Human serum typically contains more phospholipid and cholesterol, and less fatty acid than AlbuMAX (Frankland et al., 2007). AlbuMAX has several advantages over human serum for culture of *P. falciparum* due to its low cost, compatibility with any blood type, and lower batch-to-batch variation. AlbuMAX-supplemented culture medium has made significant contributions to malaria research, facilitating *in vitro* drug sensitivity assays for screening and monitoring of antimalarial drugs, parasite growth competition assays to measure fitness costs, and research on parasite molecular biology and immunology. However, several studies have found parasite growth rate differences between AlbuMAX- and human serum-based cultures and these can impact drug susceptibility profiling. For example, AlbuMAX supported parasite growth less well than human serum for clinical isolates from Cameroonian patients (Basco, 2004), and for long-term lab culture adapted parasites (Dohutia et al., 2017). Furthermore, the 50% inhibitory concentrations (IC_50_s) of multiple antimalarial drugs obtained with AlbuMAX, including CQ, amodiaquine, quinine and ART, were almost twice the corresponding values obtained with human non-immune serum (Ringwald et al., 1999).

This study is founded on the observation that parasites can show profound differences in competitive growth in serum and AlbuMAX: NF54, clone 3D7, outcompetes NHP4026, a parasite clone from Thailand (Tirrell et al., 2019), in media containing human serum, while NHP4026 outcompetes 3D7 in media containing AlbuMAX. We therefore generated replicate genetic crosses between these two parasites, and measured changes of allele frequency in independent progeny populations during parallel asexual blood stage growth in serum and AlbuMAX. This revealed three repeatable quantitative trait loci (QTL) regions, on chromosomes 2, 13 and 14, that were associated with large differences in parasite growth rates in the two medium types. Finally, using knockout parasites, we showed that an RBC invasion gene, erythrocyte binding antigen-140 (*EBA-140*), causes the largest QTL for this environment **×** genotype interaction.

## Materials and Methods

### Ethics Approval and Consent to Participate

The study was performed in strict accordance with the recommendations in the Guide for the Care and Use of Laboratory Animals of the National Institutes of Health (NIH), USA. To this end, the Seattle Children’s Research Institute (SCRI) has an Assurance from the Public Health Service (PHS) through the Office of Laboratory Animal Welfare (OLAW) for work approved by its Institutional Animal Care and Use Committee (IACUC). All of the work carried out in this study was specifically reviewed and approved by the SCRI IACUC.

### Culture Media With Serum and AlbuMAX

Contents and manufacturers of the culture media used in this study for *P. falciparum* are listed in **Supplementary Table 1**. In summary, we used RPMI 1640 as basal medium. We added 2 mM L-glutamine as amino acid supplement, 25mM HEPES for maintaining culture pH. While not essential for asexual culture (Trager and Jensen, 1976), we used 50 μM hypoxanthine as a purine precursor which helps in cell growth and gametocytogenesis. To prevent the growth of fungi and bacteria, we used 50 IU/ml penicillin, 50 µg/ml streptomycin and 50µg/ml vancomycin for the first two asexual life cycles (4 days). The basal medium was supplemented either with 10% O+ human serum (not heat-inactivated) or with 0.5% AlbuMAX II. O+ erythrocytes were added every two days into the culture media to support amplification of the parasite population. We maintained all the cultures at 37°C with 5% O_2_, 5% CO_2_, and 90% N_2_, with 5% hematocrit. Only one batch of reagents were used through the whole experiment.

### Preparation of the Genetic Cross

We generated the cross using FRG NOD huHep mice with human chimeric livers and *A. stephensi* mosquitoes as described in Vaughan et al. (Vaughan et al., 2015) and Li et al. (Li et al., 2019) (**Figure 2A**). We used NF54, clone 3D7, and NHP4026 as parental parasites. 3D7 is a clone of NF54, which was isolated from a Dutch resident who had not left the country (Delemarre and van der Kaay, 1979), and has been postulated to be of African origin (Preston et al., 2014). 3D7 was initially cloned through limiting dilution (Walliker et al., 1987). The 3D7 parasite we used for this study (and for Button-Simons et al. (Button-Simons et al., 2021) here it was referred to as NF54) was further isolated from a volunteer in a malaria clinical trial after controlled human malaria infection by 3D7-infected *Anopheles stephensi* mosquito bite (Ockenhouse et al., 1998). 3D7 has been maintained in the lab for decades, in a variety of media formulations containing both serum and AlbuMAX. NHP4026 was cloned from a patient visiting the Shoklo Malaria Research Unit (SMRU) clinic on the Thailand-Myanmar border in 2007 and has been cultured in the lab in media containing AlbuMAX for ~80 days.

Gametocytes were generated using culture media with serum from both the parasite strains and were diluted to 0.5% gametocytemia using a human serum erythrocyte mix, to generate infectious blood meals (IBMs). IBMs from each parent were mixed at equal ratio and fed to ~450 mosquitos (3 cages of 150 mosquitoes, of which ~45 mosquitoes were sacrificed for prevalence tests). Recombinants were generated after gametes fused to form zygotes in the mosquito midgut. Replication of the four meiotic products ultimately leads to the generation of thousands of haploid sporozoites within each oocyst. We examined the mosquito infection rate and oocyst number per infected mosquito 7 days post-feeding. Fifteen mosquitoes were randomly picked from each cage and dissected under microscopy. For this cross, the oocyst prevalence was 93% (range: 86-100%), with a median burden of 11 oocysts per mosquito (average of 14 oocysts per mosquito, range from 2 to 61). To ensure that recombinants from each pool were independent, we injected pooled sporozoites into each of three FRG huHep mice from a different cage of mosquitoes, each containing approximately 100 infected mosquitoes. We generated three independent recombinant pools for this experiment.

Sporozoites were isolated from individual cages containing approximately 100 infected mosquito salivary glands and 2-4 million sporozoites were injected into three FRG huHep mice (one cage per mouse), intravenously. To allow for the liver stage-to-blood stage transition, mice were infused with human erythrocytes six and seven days after sporozoite injection. Four hours after the second infusion, the mice were euthanized and exsanguinated to isolate the circulating ring stage *P. falciparum*-infected human erythrocytes. The parasites from each mouse were the initial recombinant pools used for further experiments. All initial recombinant pools were maintained using AlbuMAX culture for 24 hr to stabilize the newly transitioned ring stage parasites. We thus prepared three replicate recombinant pools for this study.

### Sample Collection and Sequencing

We aliquoted each initial recombinant pool into four cultures and maintained two with serum medium and two with AlbuMAX medium (**Figure 2B** and **Supplementary Table 1**). Cultures were set up at 1% parasitemia and 5% hematocrit with a culture volume of 5 ml per well. There were a total of 12 cultures (recombinant pools from 3 mice as biological replicates × 2 [serum + AlbuMAX] × 2 technical replicates). We maintained all the cultures in standard six-well plates for 34 days. We diluted the cultures every other day (one asexual lifecycle) to 1% parasitemia. To do this, we counted parasitemia, removed old media, cut the culture based on total parasitemia, and then added fresh erythrocytes and culture media to maintain a final volume of 5 mL. Approximately 70 µl of packed erythrocytes were collected and frozen down every 2-4 days, when cutting the culture, for downstream extraction of genomic DNA for further analysis. We extracted and purified genomic DNA using the Qiagen DNA mini kit, and quantified DNA mass using Qubit. For DNA samples with less than 150 ng (15/136 samples), we performed selective whole genome amplification (sWGA) (Li et al., 2019) before preparing libraries. Previous work has shown that this does not bias allele frequency measurement in parasite pools (Ting et al., 2005). We constructed next generation sequencing libraries using 50-100 ng DNA or sWGA/WGA product following the KAPA HyperPlus Kit protocol with 3-cycles of PCR. All libraries were sequenced using 150 bp pair-end reads to a minimum coverage of 100× using Illumina Novaseq S4 or Hiseq X sequencers.

### Genotype Calling

We genotyped the parental strains and bulk populations as described (Li et al., 2019). We mapped the whole-genome sequencing reads both from parental parasites and progeny against the reference genome (PlasmoDB version 46) using BWA mem (http://bio-bwa.sourceforge.net/) under the default parameters. We excluded the high variable genome regions (subtelomeric repeats, hypervariable regions and centromeres) and only performed genotype calling in the 21 Mb core genome (defined in (Miles et al., 2016)). The resulting alignments were then converted to SAM format, sorted to BAM format, and deduplicated using picard tools v2.0.1 (http://broadinstitute.github.io/picard/). We used Genome Analysis Toolkit GATK v3.7 (https://software.broadinstitute.org/gatk/) to recalibrate the base quality score based on a set of verified known variants (Miles et al., 2016). We called variants using HaplotypeCaller and then merged using GenotypeGVCFs with default parameters except for sample ploidy 1.

We only applied filters to the GATK genotypes of parental parasites, using standard filter methods described by McDew-White et al. (Mcdew-White et al., 2019). The recalibrated variant quality scores (VQSR) were calculated by comparing the raw variant distribution with the known and verified *Plasmodium* variant dataset, and loci with VQSR less than 1 were removed from further analysis. After filtration, we selected SNP loci that are distinct in two parents, and only used those for further bulk segregant analysis.

### Bulk Segregant Analysis

We performed the bulk segregant analysis as described before (Li et al., 2019). Only loci with coverage > 30× were used for bulk segregant analysis. We counted reads with genotypes of each parent and calculated allele frequencies at each variable locus. Allele frequencies of 3D7 were plotted across the genome, and outliers were removed following Hampel’s rule (Davies and Gather, 1993) with a window size of 100 loci. We performed the bulk segregant analyses using the R package QTLseqr (Mansfeld and Grumet, 2018). QTLs were defined as regions with G’ > 20 (Magwene et al., 2011). Once a QTL was detected, we calculated and approximate 95% confidence interval using Li’s method (Li, 2011) to localize causative genes. We also measured the fitness cost at each mutation by fitting a linear model between the natural log of the allele ratio (freq[allele1]/freq[allele2]) against time (measured in 48hr parasite asexual cycles). The slope provides a measure of the selection coefficient (*s*) driving each mutation (Dykhuizen and Hartl, 1980). The raw *s* values were tricube-smoothed with a window size of 100 kb to remove noise (Nadaraya, 1964; Watson, 1964).

### Head-to-Head Competition Experiments

We conducted head-to-head competition experiments between parental 3D7 and NHP4026 parasites, and between the 3D7Δ*^EBA-140^* and the 3D7 wildtype parasites. For each comparison, we synchronized the parasites at ring stages. These parasites were adapted to respective media formulations for two asexual replication cycles. The two competitor parasites were then mixed in a 1:1 ratio at 1% ring stage parasitemia and 5% hematocrit and cultured as two technical replicates in the respective media formulations. We maintained two of the aliquots in human serum supplemented media, two in AlbuMAX supplemented media, and two in medium containing equal amount of serum and AlbuMAX. All competition assays were conducted in 6-well plates, with a 5 ml culture volume. All cultures received media changes every day and cultures were cut to 1% parasitemia every other replication cycle. 50 µl of packed erythrocytes were collected every four days to monitor the outcome of each competition and frozen at -80°C until genomic DNA was extracted.

For the 3D7 vs NHP4026 competition, we used a microsatellite-based method developed by Tirrell et al. (Tirrell et al., 2019). PCR amplification products from microsatellite TA119 (forward primer: TCCTCGATTATATTATTGCA; reverse primer: TAATACATTCCCATTAGATC) were analyzed using Applied Biosystems 3730xI DNA Analyzer (ThermoFisher). Relative density of amplicons from each parent was then scored by the height of corresponding fluorescent peak comparing to the overall signal.

For the 3D7 vs 3D7Δ*^EBA-140^* competition, we used a qPCR assay to determine the frequency of each parasite. The 3D7Δ*^EBA-140^* parasites were generated by Maier et al., using transfection plasmid pHH1ΔEBA140 containing the human dihydrofolate reductase gene (*hDHFR*) (Maier et al., 2003). The plasmid was integrated into the genome in the creation of the 3D7Δ*^EBA-140^* parasite and is absent from wildtype 3D7 parasites, and thus delineates the 3D7Δ*^EBA-140^* and wildtype 3D7 parasite genome. We thus used a qPCR-based method to determine the allele frequency of 3D7Δ*^EBA-140^* and 3D7 parasites: we designed qPCR primers (*p1*-forward: CGGAATGGCGAATAACAATG; *p1*-reverse: ATTCCCCGCACTTTCCTCTT) to amplify genomic regions for both 3D7Δ*^EBA-140^* and wildtype parasites; we designed another set of primers (*p2*-forward: TGATGTCCAGGAGGAGAAAGG; *p2*-reverse: TCGCTATCCCATAAATTACAAAACA) which covers the junction between *hDHFR* gene and a short sequence within the pHH1ΔEBA140 plasmid, to amplify genome regions only from 3D7Δ*^EBA-140^* parasites. Both *p1* and *p2* primer sets did not amplify a PCR product from human DNA.

We ran two qPCR reactions for each sample separately, using the two different sets of primers (*p1*-forward with *p1*-reverse and *p2*-forward with *p2*-reverse). The qPCR reaction mixture contained 5 µL of SYBR Green MasterMix (Applied Biosystems, Carlsbad, California, USA), 0.3 µL of each 10 µM primer stock (total of 0.6 µL), 3.4 µL of sterile water and 1 µL of total DNA template. The qPCR reactions were performed in duplicate using the AB1 prism 7900HT Sequence Detection system (Applied Biosystems, Carlsbad, California, USA) as follows: 95°C for 10 min, then 40 cycles of 95°C for 15 sec and 60°C for 1 min. We examined the melting curve (60–95°C) at the end of each assay to verify the uniqueness of the PCR products generated. We plotted standard curves using artificial genome DNA mixtures of 3D7Δ*^EBA-140^* and wildtype parasites (ratio at 1:0, 0.25:0.75, 0.5:0.5, 0.75:0.25 and 0:1). The 3D7Δ*^EBA-140^* allele frequency was then calculated as copies of 3D7Δ*^EBA-140^* parasite genomes/copies of total parasite genomes.

We calculated selection coefficients for each allele using the formula as described above in the **Bulk segregant analysis** section. Positive values of *s* indicated a selective disadvantage for NHP4026 or 3D7Δ*^EBA-140^* alleles.

## Results

### The Outcome of Parasite Competition Is Reversed in Different Media

**Figure 1:**
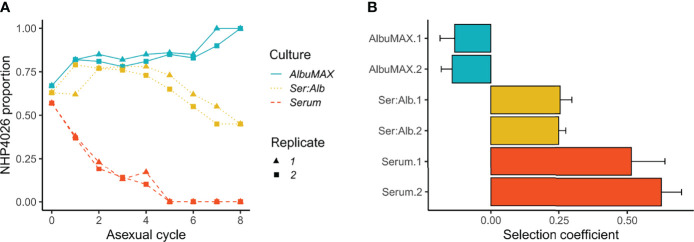
Outcome of competition between 3D7 and NHP4026 under different culture conditions. **(A)**. Proportion of NHP4026 at each parasite asexual cycle. **(B)**. NHP4026 selection coefficients (*s*) with 1× standard error. Positive *s* values indicate a disadvantage for alleles inherited from NHP4026. Culture conditions: AlbuMAX, medium contained only AlbuMAX; Ser:Alb, medium contained serum and AlbuMAX at a 50:50 ratio; Serum, culture medium contained only serum.

We measured the competitive fitness of 3D7 and NHP4026 blood stage parasites in media containing human serum, AlbuMAX, or a combination of these medium types (**Figure 1**). 3D7 outcompeted NHP4026 in media containing human serum, while NHP4026 outcompeted 3D7 in media containing AlbuMAX, and the two parasites showed comparable fitness in media containing both human serum and AlbuMAX (**Figure 1A**). The fitness differences observed in human serum and AlbuMAX were large. 3D7 replaced NHP4026 in five asexual life cycles in media containing serum: this resulted from a selective advantage (*s*) of 0.51 ± 0.15 (1× standard error) (replicate 1) and 0.62 ± 0.07 (replicate 2) per 48 h asexual cycle (**Figure 1B**). In contrast, NHP4026 replaced 3D7 in eight asexual life cycles in AlbuMAX, and the relative fitness was reversed (*s* = -0.13 ± 0.05 for replicate 1 and -0.14 ± 0.04 for replicate 2) (**Figure 1B**). The differences in fitness observed between the two medium types were highly significant (*p* = 7.37E-10).

### Genetic Crosses and Composition of Segregant Pools

**Figure 2:**
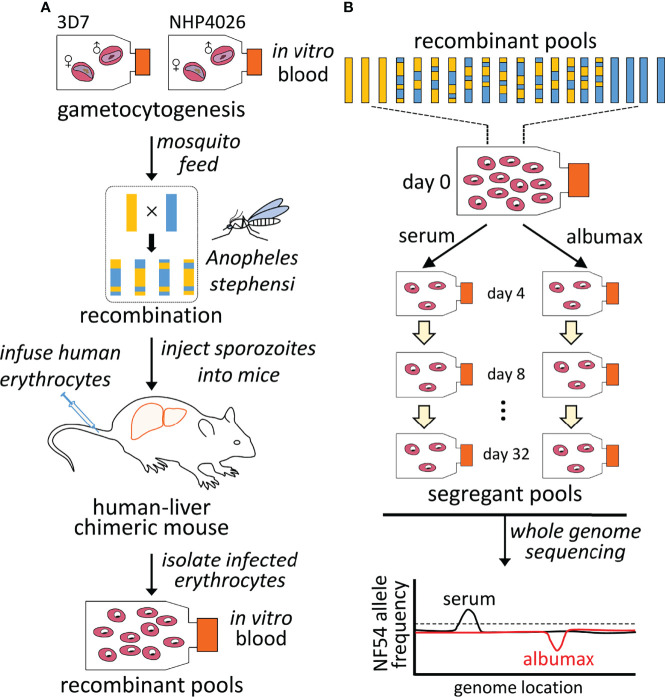
Mapping parasite fitness under different culture conditions. **(A)**. Recombinant progeny pool generation. Genetic crosses were generated using female *Anopheles stephensi* mosquitoes and FRG huHep mice as described by Vaughan, 2015. Recombination of parasite genomes occurs during meiosis in the mosquito midgut. Recombinant pools were collected from infected mice and maintained in *in vitro* asexual blood stage culture. **(B)**. Bulk segregant analysis. Pools of progeny were cultured in parallel with serum or AlbuMAX and samples were collected every four days. Each segregant pool was then whole-genome sequenced and genotyped in bulk. Differences in allele frequency among different groups were used to identify QTLs.

We generated a genetic cross between 3D7 and NHP4026 (**Figure 2A**). A total of 13,195 single nucleotide polymorphisms (SNPs, **Supplementary Table 2**) differentiate the two parental parasites within the 21 Mb core genome (defined in (Miles et al., 2016)). We used *Anopheles stephensi* mosquitoes and FRG huHep mice (Vaughan et al., 2015) to generate three independent recombinant pools. Combining all the information, we estimate that there were 28 (average 14 oocysts per mosquito × 4 recombinants per oocyst × 0.5 selfed) recombinant genotypes per infected mosquito, which gave us ~2800 (28 × ~100 infected mosquitoes) unique recombinants per pool for each of the three mice (see *Preparation of the Genetic Cross*). In addition to the bulk segregant analyses described below, we cloned and carried out whole genome sequencing (WGS) of progeny from these recombinant pools using published methods (Button-Simons et al., 2021) from a mixture of all pools a week after transferring the transitioned blood stages to *in vitro* culture. This revealed low numbers of selfed progeny (2/55) in this cross with very little redundancy among recombinants (**Supplementary Table 3**), as observed in previous crosses (Button-Simons et al., 2021). Based on bulk segregant analyses of the initial recombinant pools, the allele frequencies of 3D7 emerging from the liver were 0.52 ± 0.001, 0.50 ± 0.002 and 0.55 ± 0.002 for the three mice. We cultured each recombinant pool with O-positive non-immune human serum or AlbuMAX for 34 days in parallel, with two technical replicates in each medium type for each of the three biological replicates. We collected samples for bulk segregant analysis every four days and used WGS to analyze all segregant pools to high (191× ± 40 read depth) genome coverage (**Figure 2B**).

### Bulk Segregant Analysis Identifies QTLs for Differential Growth in Human Serum and AlbuMAX

**Figure 3:**
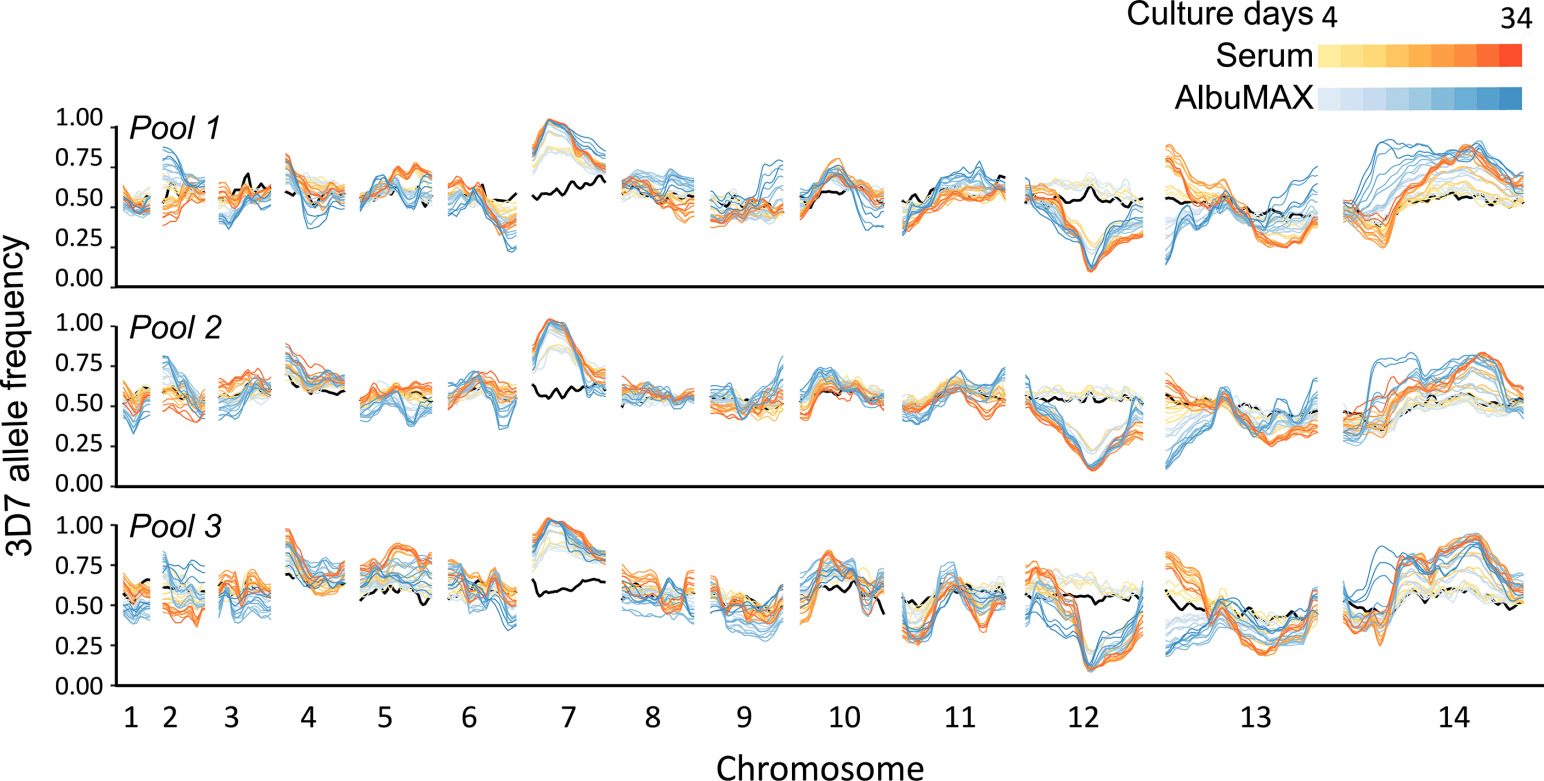
Change in genome frequency under different culture conditions. The black lines show allele frequencies from the initial recombinant pools. Yellow-red lines show frequency changes in serum over time and light blue-dark blue lines show frequency changes over time in or AlbuMAX. The three plots show allele frequencies from the three recombinant pools (biological replicates). The lines with the same color in each panel show values for the two technical replicates.

**Figure 4:**
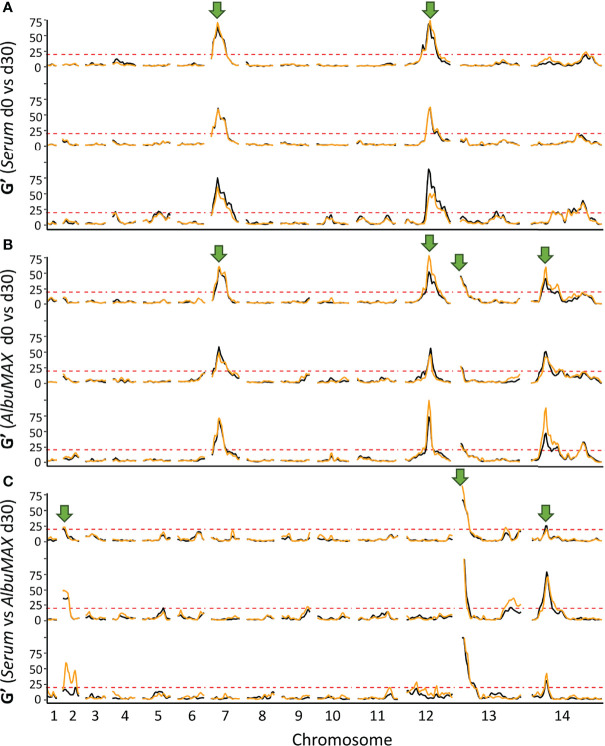
QTLs defined with the G’ approach. The top **(A)** and middle **(B)** panels show QTLs detected by comparing allele frequencies from the initial recombinant pools and pools after 30 days of serum (top) or AlbuMAX (middle) culture. The bottom panel **(C)** indicates QTLs at day 30 when comparing serum and AlbuMAX cultures. There are three plots in each panel, representing analyses from the three recombinant pools (biological replicates). Orange and black lines delineate technical replicates in each experiment. We used a threshold (G’ > 20) to determine significant QTLs. Arrows mark the position of QTLs identified.

**Figure 5:**
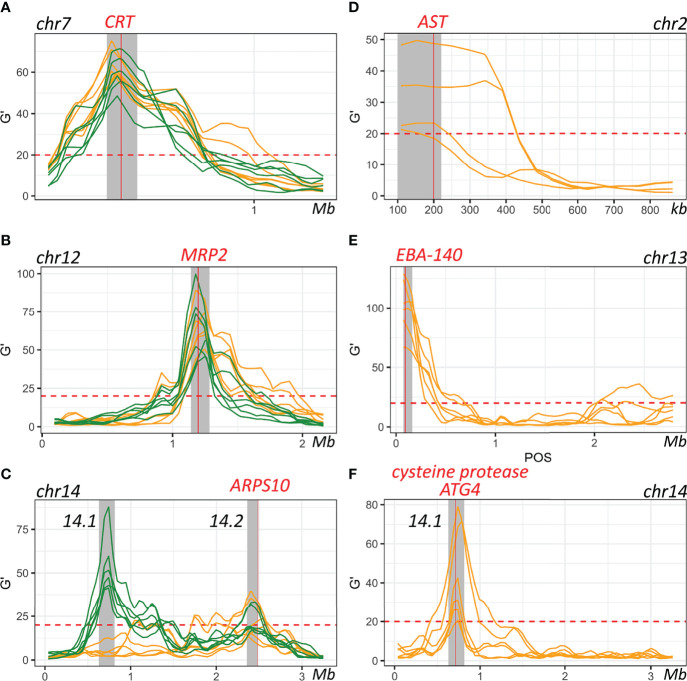
Genes within defined QTL regions. **(A–C)**. QTLs detected through comparing allele frequencies from the initial recombinant pools and pools after 30 days of serum (orange) or AlbuMAX (green) culture. **(D–F)**. QTLs detected by comparing serum and AlbuMAX after 30 days of culture. Each line is one comparison. Grey shadows indicate boundaries of the merged 95% confidential intervals (CIs) for each QTL.

By comparing allele frequency changes in human serum and AlbuMAX culture over 34 days (**Figure 3**), we detected three QTLs (**Table 1**, **Figure 4**, **Supplementary Figure 1**) situated at the beginning of chromosome 2 (named QTL chr. 2), the beginning of chromosome 13 (QTL chr. 13) and the first half of chromosome 14 (QTL chr. 14.1). For each detected QTL (G’ > 20), we calculated 95% confidence intervals to narrow down the size of the genome region and thus the list of genes that could be driving selection (**Figure 5**). The list of genes inside the QTL regions is summarized in **Supplementary Table 4**. We prioritized genes within these genome regions by the following criteria: i) evidence of gene expression in blood stages; ii) gene annotations and related metabolic pathways; iii) inspection of SNPs and indels that differentiated the two parents (**Supplementary Table 5**, **6**). The strongest candidate genes driving these QTLs are listed in **Table 1**.

**Table 1:** Top candidate genes located inside of the QTL regions.

QTL ID	QTL*^a^*	95% CI*^b^*	Gene ID	Gene name	Gene annotation
*QTLs for differential growth in serum and AlbuMAX*					
chr. 2	serum vs AlbuMAX	chr2: 0 – 220,805	PF3D7_0204500	*AST*	aspartate transaminase
chr. 13	serum vs AlbuMAX	chr13: 0 – 162,844	PF3D7_1301600	*EBA-140*	erythrocyte binding antigen-140
chr. 14.1	serum vs AlbuMAX	chr14: 629,528-812,881	PF3D7_1417300	*ATG4*	cysteine protease ATG4
*QTLs revealed by allele frequency skews in both serum and AlbuMAX*					
chr. 7	serum and AlbuMAX	chr7:340,864 - 476,223	PF3D7_0709000	*CRT*	chloroquine resistance transporter
chr. 12	serum and AlbuMAX	chr12:1,141,368 - 1,282,609	PF3D7_1229100	*MRP2*	multidrug resistance-associated protein 2
chr. 14.2	serum and AlbuMAX	chr14:2,356,428 - 2,485,473	PF3D7_1460900	*ARPS10*	apicoplast ribosomal protein S10

^a^, serum vs AlbuMAX QTLs were identified by comparing differential growth in human serum and AlbuMAX; serum/AlbuMAX QTLs were detected in both Serum and AlbuMAX.

^b^, 95% confidence interval (CI) using Li’s method (Li, 2011).

For the chr. 2 QTL, the 3D7 allele frequency increased over time in AlbuMAX and decreased in serum; while the opposite pattern was observed for the chr. 13 QTL. The patterns of divergent selection observed were consistent across all biological and technical replicates (**Supplementary Figure 2** and **Supplementary Table 7**). For 3D7, we observed *s* = 0.04 ± 0.01 in serum and *s* = -0.06 ± 0.01 in AlbuMAX for the chr. 2 QTL region; *s =* -0.08 ± 0.02 in serum and *s =* 0.15 ± 0.01 in AlbuMAX for chr. 13. These two QTL regions are located at the beginning of the chromosome. It was difficult to define QTL boundaries to a narrow confidence interval due to limited recombination and difficulties of sequence alignment in these regions. For the chr. 2 QTL, we inspected genes located from the beginning of chr. 2 to 220 kb (chr. 2 has a length of 947 kb) (**Figure 5**). This region contained 53 genes: 14 are not expressed in blood stage parasites and were excluded, and 13 of the remaining 39 were considered high priority candidates. Among these, the aspartate transaminase gene (*AST*, PF3D7_0204500, also known as aspartate aminotransferase, *AspAT*), is a critical enzyme for amino acid metabolism (Toney, 2014). There were no non-synonymous mutations in the coding region in the *AST* alleles from the two parents, but we found multiple differences within the 5’ UTR and gene expression regulatory regions, including two SNPs (c.-18C>T [18 bp upstream the coding region] and c.-29A>C) and three microsatellites (**Supplementary Table 5**).

The chr. 13 QTL contained 33 genes (23 expressed in blood stage, 8 high priority candidates) and spanned 163 kb at the beginning of chr. 13. Among the genes in this QTL was erythrocyte binding antigen-140 (*EBA-140*, PF3D7_1301600). EBA-140 mediates *P. falciparum* RBC invasion by binding to the RBC receptor glycophorin C and initiating merozoite entry (Adams et al., 1992) and expression levels are variable in parasites from different clinical isolates (Gomez-Escobar et al., 2010). Interestingly, there is a single non-synonymous mutation (Leu112Phe) between 3D7 and NHP4026. This SNP is located before the first Duffy-binding-like (DBL) domain (Lin et al., 2012) and is common in malaria populations (**Supplementary Figure 3**). The *EBA-140* allele from NHP4026 was preferentially selected for in media containing AlbuMAX (**Supplementary Figure 2**).

In the chr. 14.1 QTL region, the 3D7 allele frequency did not change over time during growth in serum but increased significantly during growth in AlbuMAX (**Figure 3**). The selection coefficient *s* = 0.02 ± 0.02 in serum and *s* = -0.10 ± 0.03 in AlbuMAX (**Supplementary Figure 2**). This QTL, located in the first half of chr. 14 (630 kb – 813 kb, **Supplementary Table 6**), spanned 183 kb and contained 38 genes. Of these genes, 37 are expressed in blood stages, and 11 are high priority candidates. Interestingly, NHP4026 carried a single amino acid deletion (Asn226del) and three non-synonymous mutations (Ser329Pro, Asn503Lys and Val556Ile) in the cysteine protease *autophagy-related 4* gene (*ATG4*, PF3D7_1417300) within this region.

### Functional Validation of *EBA-140* as the Causative Gene Within the chr. 13 QTL

**Figure 6:**
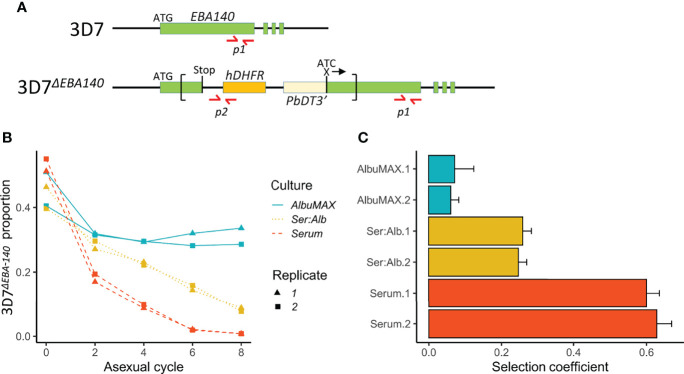
Outcome of competition between 3D7 and 3D7Δ*^EBA-140^* under different culture conditions. **(A)** Design of qPCR primers. Top, structure of the 3D7 *EBA-140* gene locus; bottom, disrupted *EBA-140* gene locus in 3D7Δ*^EBA-140^*. The recombinant plasmid pHH1ΔEBA-140 (sequence shown inside the square brackets, was integrated into the *EBA-140* gene during the generation of 3D7Δ*^EBA-140^* (Maier et al., 2003). The locations of the qPCR primers are labeled with red arrows. Amplicon *p1* (arrows indicate forward and reverse primers) is located at the 3’ end of the *EBA-140* gene and amplifies DNA from both 3D7 and 3D7Δ*^EBA-140^*. Amplicon *p2* was designed to cover the junction between the human dihydrofolate reductase gene (*hDHFR*) (orange box) and a short upstream sequence within the pHH1ΔEBA140 plasmid and can only be amplified from 3D7Δ*^EBA-140^*. **(B)** The proportion of 3D7Δ*^EBA-140^* over eight parasite asexual cycles. **(C)** Selection coefficients (*s*) of 3D7Δ*^EBA-140^* with 1× standard error. Positive values of *s* indicate a disadvantage to 3D7Δ*^EBA-140^*. Culture conditions: Ser:Alb culture media contains both serum and AlbuMAX in a 50:50 ratio.

We chose the strongest QTL on chr. 13 for functional analysis. To do this, we utilized *EBA-140* gene disrupted parasites generated in 3D7 (3D7Δ^EBA-140^) (Maier et al., 2003). 3D7 is a clonal line (Walliker et al., 1987), and one of the parents in our genetic cross, so provides an appropriate genetic background for interrogating the role of *EBA-140*. We conducted head-to-head competition experiments between 3D7Δ^EBA-140^ and 3D7 wildtype parasites in media containing serum, AlbuMAX or a serum/AlbuMAX mixture to measure fitness consequences of *EBA-140* disruption. We then used qPCR to quantify frequencies of the two competing parasite lines in each experiment (**Figure 6**). 3D7Δ^EBA-140^ showed lower fitness than wildtype parasites in all experiments, but the impact of *EBA-140* disruption on fitness was strongly dependent on media composition. Fitness costs of 3D7^ΔEBA-140^ were low in AlbuMAX (*s* = 0.07 ± 0.04) intermediate in serum: AlbuMAX mixtures (*s* = 0.25 ± 0.02) and high in serum (*s* = 0.61 ± 0.04). Fitness costs resulting from the ΔEBA-140 were significantly higher in serum than in AlbuMAX (Δ*s* = 0.55, *p* = 5.0E-08), suggesting that *EBA-140* is the causative gene within the chr. 13 QTL.

### Systematic Skews Observed in Both Serum and AlbuMAX

We observed three genome regions that showed strong distortions in allele frequency in each independent replicate cross in both human serum and AlbuMAX cultures (**Figure 3**), consistent with our earlier report (Li et al., 2019; Button-Simons et al., 2021): on chr. 7 (named QTL chr. 7), in the middle of chr. 12 (QTL chr. 12) and on the second half of chr. 14 (QTL chr. 14.2). We observed strong selection against NHP4026 alleles in the chr. 7 and chr. 14 QTL regions (*s* = 0.29 ± 0.04 in serum and 0.26 ± 0.04 in AlbuMAX for chr. 7, *s* = 0.12 ± 0.02 in both serum and AlbuMAX for chr. 14.2), while 3D7 alleles were selected against in the chr. 12 QTL region, with *s* = 0.30 ± 0.05 in serum and 0.25 ± 0.06 in AlbuMAX (**Supplementary Figure 2**). The skews were consistent among all three biological replicates.

The chr. 7 QTL spanned from 341 kb to 476 kb (135 kb) and contained 33 genes (**Supplementary Table 4**). *PfCRT* (PF3D7_0709000), which is known to carry a high fitness cost associated with CQ resistant alleles (Ecker et al., 2012), is located at the peak of the chr. 7 QTL (**Figure 5**). Here, NHP4026 carries the CQ resistant *PfCRT* allele while 3D7 is CQ sensitive (**Supplementary Table 7**). In both serum and AlbuMAX cultures, the resistant CQ allele carries an extremely high fitness cost (*s* = 0.29 ± 0.04 in serum and *s* = 0.26 ± 0.04 in AlbuMAX) and is evident as early as day four of *in vitro* culture (**Supplementary Figure 2**).

The QTL on chr. 12 spanned from 1,141 kb to 1,283 kb (142 kb) and contained 32 genes all expressed in blood stage, of which 9 are high priority candidates. The QTL on chr. 14 (numbered 14.2 in **Table 1** and **Supplementary Table 4**) spanned from 2,356 kb to 2,485 kb (129 kb) and contained 23 genes, of which 10 are high priority candidates. These same regions carried high fitness costs in a bulk segregant analysis from an independent genetic cross between two different parental parasites – ART-sensitive MKK2835 and ART-resistant NHP1337 (Li et al., 2019). The multidrug resistance-associated protein 2 (*MRP2*, PF3D7_1229100) and apicoplast ribosomal protein S10 (*ARPS10*, PF3D7_1460900), are both located inside of the chr. 12 and chr. 14.2 QTL regions respectively. There are total of four non-synonymous mutations and six indels within the *MRP2* locus, and all four parental parasites carry different *MRP2* alleles. *MRP2* alleles from 3D7 (from this study) and NHP1337 (from Li et al. (Li et al., 2019)) carried high fitness costs during *in vitro* blood cultures with serum containing media. There were two non-synonymous mutations (Val127Met and Asp128His) in the ARPS10 locus (**Supplementary Table 5**). *ARPS10* alleles with these two mutations (NHP4026 in this study and NHP1337 in previous study by Li et al. (Li et al., 2019)) carry a high fitness cost during *in vitro* culture with serum. The Val127Met mutation is one of the genetic background SNPs for *kelch13* alleles on which ART resistance emerged in Cambodia (Miotto et al., 2015).

## Discussion

### Parasite Growth Rates and Fitness Are Dependent on Growth Media

A central finding of this work is that parasite fitness in blood stage culture is strongly dependent on the growth media used. The outcome of competition experiments between the two parental parasites were completely reversed depending on whether media contained serum or AlbuMAX as a lipid source. Surprisingly, the newly isolated parasite, NHP4026, shows high fitness in AlbuMAX, while 3D7, which has a long history of laboratory cultivation using both serum and AlbuMAX based media, shows highest fitness in media containing serum. The fitness traits observed do not appear to be driven by short-term adaptation and are segregating in the progeny of the genetic crosses, indicative of a genetic basis. The strong association between fitness and growth environment has important implications for experimental malaria biology. Fitness-based assays are widely used for examining the impact of piggyBac insertions (Zhang et al., 2018), determining the fitness costs of antimalarial drug resistance (Nair et al., 2018; Stokes et al., 2021), and measuring red blood invasion (Ebel et al., 2021). These results add weight to previous observations that serum or AlbuMAX based media can impact measurements of drug resistance (Ringwald et al., 1999), and presentation of antigens on the RBC surface (Frankland et al., 2007). Our observation that parasite fitness is dependent on culture conditions highlights previous reviews on the disagreements between *in vitro* and *in vivo* parasite phenotypes (Leroux et al., 2009; Brown and Guler, 2020).

### Locus Specific Selection Between Serum and AlbuMAX Cultures

Because the parental parasites showed distinctive patterns of growth in serum or AlbuMAX, we were able to use genetic crosses, in combination with bulk segregant analysis to determine the genome regions underlying differential growth in these two medium types. We found three QTL regions where allele frequencies of recombinant progeny parasite pools showed dramatic divergence, depending on whether they were grown in serum or in AlbuMAX (**Figure 4**). QTLs for differential selection in serum and AlbuMAX were observed in each of the biological replicate recombinant pools and across technical replicates for each pool. Furthermore, selection driving change in allele frequency in these three QTL regions is strong (*s* = 0.10 ± 0.01 [chr. 2], *s* = 0.23 ± 0.02 [chr. 13] and *s* = 0.12 ± 0.02 [chr. 14.1], **Table 1**).

AlbuMAX is a lipid-loaded bovine serum albumin, while the composition of serum is more complex, containing a variety of proteins and peptides (albumins, globulins, lipoproteins, enzymes and hormones), nutrients (carbohydrates, lipids and amino acids), electrolytes, and small organic molecules. Serum also contains more phospholipid and cholesterol and less fatty acid than AlbuMAX (Frankland et al., 2007). It has been previously reported that AlbuMAX doesn’t support malaria parasite growth as well as serum (Ringwald et al., 1999; Basco, 2004; Dohutia et al., 2017). Our analysis reveals three QTLs across the genome that determine parasite growth rate in serum versus AlbuMAX, indicating that this trait has a relatively simple genetic basis in this cross, and suggesting that these QTL regions contain genes involved in nutrient uptake, metabolism, or interactions with serum components. By inspection of the genes under these QTL peaks, we identified three genes – *AST* (QTL chr. 2), *EBA-140* (chr. 13) and cysteine protease *ATG4* (chr. 14.1) – as the strongest candidates (**Table 1**, full gene listed in **Supplementary Table 6**).

### Interplay Between RBC Invasion and Growth Media

These experiments were initially conceived to identify genes that underlie nutrient utilization/metabolism in *P. falciparum*. However, our functional analysis of the largest QTL (chr. 13) reveals that *EBA-140*, a gene known to be involved in RBC invasion (Maier et al., 2003), underlies differential growth in media containing human serum or AlbuMAX. EBA-140 mediates *P. falciparum* RBC invasion by binding to the RBC receptor glycophorin C (Adams et al., 1992). These results suggest that there are intimate links between the efficiency of RBC invasion and media composition. How might such links be mediated? One possibility is that the culture condition could alter the expression of the EBA-140 protein. A previous study has shown that surface expression of PfEMP1 was reduced in AlbuMAX medium compared to serum (Frankland et al., 2007). Furthermore, mutations in *EBA-140* influence parasite ligand-binding to RBC receptors (Adams et al., 1992; Lin et al., 2012). In our study, only one amino acid change (Leu112Phe) distinguishes *EBA-140* from 3D7 and NHP4026. We speculate that this mutation influences EBA-140 binding to its host RBC receptor, but that the impact of this mutation is strongly dependent on media-induced changes in the conformation, abundance or accessibility of receptors on the RBC surface, or the function of EBA-140 itself during the process of merozoite invasion. While the knockout studies conducted here strongly suggest involvement of EBA-140, allelic swap experiments, in which the Leu112Phe variant is reversed on each of the parental backgrounds, will be required to definitively determine involvement of this amino acid, to investigate the role of EBA-140 gene expression and protein abundance and to further understand the molecular basis for this interesting phenotype.

### Candidate Genes Driving the chr. 2 and chr. 14.1 QTLs

Some compelling candidate loci that may drive differential growth in serum or AlbuMAX were also found under the two smaller QTLs:

Chr. 2: *P. falciparum* acquires nutrients from the host through catabolism of hemoglobin in erythrocytes. During this process, plasmodial AST plays a critical role in the classical tricarboxylic acid (TCA) cycle, and also functions to maintain homeostasis of carbohydrate metabolic pathways (Wrenger et al., 2012). AST is essential (Ting et al., 2005), which highlights this gene as a potential bottleneck in energy metabolism and as a target for the design of novel therapeutic strategies. We speculate that selection acts on the regulation of *AST* gene expression levels: we detected no non-synonymous mutations in the coding region, but we found multiple variants in the 5’ UTR and regulatory regions (**Supplementary Table 5**). However, we cannot exclude the possibility that other neighboring loci may drive the observed allele frequency changes.

Chr. 14.1: Cysteine protease ATG4 is essential for autophagy in both yeast, mammals, and protozoan parasites such as *Leishmania major* and *Trypanosoma cruzi* (Siqueira-Neto et al., 2018). Interestingly, the *Plasmodium* genome does not encode for all the components of a canonical autophagy pathway (PlasmoDB), and although *P. falciparum ATG4* is amenable to mutation, mutant parasites showed a very high fitness cost in the asexual blood stage, pointing to an essential role in parasite survival (Zhang et al., 2018). Autophagy involves vesicular trafficking and is important for protein and organelle degradation during cellular differentiation (Levine and Klionsky, 2004). In our study, the 3D7 *ATG4* allele was associated with fitness during AlbuMAX culture, whereas allele frequencies did not change in serum (**Supplementary Figure 2**). As AlbuMAX contains fewer nutritional components than serum (Lin et al., 2012), we speculate that parasites may require higher levels of autophagy to compensate for specific nutrients that are missing in AlbuMAX and the 3D7 allele fulfills this role. In addition malaria parasites have several cysteine proteases with functions including hemoglobin hydrolysis, RBC rupture, and RBC invasion (Rosenthal, 2004): two of these (serine repeat antigen 3 and 4) show >2-fold higher expression in AlbuMAX supplemented media (Singh et al., 2007).

### Replication of Fitness-Related QTLs in Independent Genetic Crosses

We detected three additional regions of the genome (QTL chr. 7, chr. 12 and chr. 14.2) that show extreme skews in allele frequencies in both serum and AlbuMAX cultures: (i) Chr. 7. The strong chr. 7 QTL is almost certainly driven by selection against the CQ resistant (CQR) *PfCRT* allele from NHP4026. The same skew was also observed in clones isolated from our previous crosses between the same parental parasites (Button-Simons et al., 2021). Interestingly, NHP4026, the parent carrying the CQR *PfCRT* allele, shows high competitive fitness in AlbuMAX, ranking above other SE Asia clinical isolates (Tirrell et al., 2019), and even outcompetes 3D7, which carries the CQ sensitive (CQS) *PfCRT* allele. We speculate that NHP4026 carries compensatory loci that restore fitness in this CQR parasite, but that recombination decouples alleles at *PfCRT* and compensatory loci, resulting in the high fitness cost of *PfCRT* CQR alleles observed in our crosses. (ii) Chr. 12. In previous genetic crosses using different parental parasites (Li et al., 2019) and in the current study, multidrug resistance-associated protein 2 (MRP2) was located at the peak of the chr. 12 QTL. *MRP2* belongs to the C-family of ATP binding cassette (ABC) transport proteins that are well known for their role in multidrug resistance. MRP2 mediates the export of drugs, toxins, and endogenous and xenobiotic organic anions (Nies and Keppler, 2007), which can thus lead to resistance to multiple drugs. MRP2 may also contribute to the detoxification of antimalarial drugs. Further experiments are needed to directly determine the function of MRP2 in parasite fitness during *in vitro* culture. (iii) Chr. 14.2. This QTL contains the *ARPS10* gene, which is thought to provide a permissive genetic background for the emergence of ART resistance (Miotto et al., 2015) (**Supplementary Table 8**): this QTL was seen both in this study and our previous cross (Li et al., 2019). Further studies are required to determine whether the mutant *ARPS10* allele might compensate for fitness costs associated with mutant *kelch13* through epistatic interactions.

### Perspectives on Bulk Segregant Approaches for Identifying RBC-Stage Growth-Related Genes

Our study uses a bulk segregant analysis strategy to systemically identify genes involved in competitive growth, nutrient uptake and metabolism. In contrast, conducting these analyses in a traditional framework, using isolation of individual progeny, measurement of growth phenotypes of each progeny in different media, and genomic characterization of progeny and parents, as required for previous nutritional genetics experiments with *P. falciparum* (Nguitragool et al., 2011; Gupta et al., 2018; Gupta et al., 2020) is laborious and time consuming. A particular advantage of bulk segregant analysis is that selections are applied to all recombinant progeny simultaneously in a single culture, removing batch effects and experimental variation resulting from conducting parallel measures with individual progeny clones. However, we note that bulk segregant analysis cannot determine the interactions among loci: this can be approached by examining growth of cloned progeny carrying different combinations of parental alleles at the three QTLs.

In this particular experiment, we compared media containing different lipid sources. The chemical differences between serum and AlbuMAX are complex, so it will be difficult to identify the precise media components driving differential selection. However, following the example of bacterial genetics where selective media has played a central role (Ryan, 1946; Shuman and Silhavy, 2003; Maheshwari, 2011), we envisage that screening of parental parasites in media that differ in levels of single nutrients will identify parasites with differing nutrient requirements. Subsequent genetic crosses and bulk segregant analysis experiments can then localize the genes underlying these nutritional polymorphisms. Such experiments can be further extended to examine RBC invasion phenotypes by conducting bulk segregant analysis experiments using RBCs carrying different invasion receptors: such RBCs can now be efficiently generated using CRISPR/Cas9 editing of hemopoietic stem cells (Scully et al., 2019), so other aspects of host genetics can be maintained. We conclude that genetic crosses and bulk segregant analysis can provide a versatile approach for dissecting the genetic basis of nutrient acquisition, and RBC invasion in determining parasite growth, and for examining host parasite interactions at the molecular level.

## Data Availability Statement

The datasets presented in this study can be found in online repositories. The names of the repository/repositories and accession number(s) can be found in the article/**Supplementary Material**.

## Ethics Statement

The animal study was reviewed and approved by IACUC, Seattle Children’s Research Institute, Seattle, Washington.

## Author Contributions

SK, XL, AMV, and TJCA designed the experiments. SK, MTH, BAA, SYK and NC prepared the crosses and collected samples. MM-W, AR, ED, and AS prepared the genomic DNA libraries. FN provided the parental parasite, NHP4026, from Thailand. LAC cloned the parental parasites. XL performed all the NGS analysis and data curation. SK, XL, and TJCA wrote the original manuscript. KVB, KAB-S, IHC, SHIK, FN, MTF, and AMV reviewed and edited the manuscript. All authors contributed to the article and approved the submitted version.

## Funding

This work was supported by National Institutes of Health (NIH) program project grant P01 AI127338 (to MF), by NIH grant R37 AI048071 (to TJCA) and NIH grant R21 AI133369 (to AV). Work at Texas Biomedical Research Institute was conducted in facilities constructed with support from Research Facilities Improvement Program grant C06 RR013556 from the National Center for Research Resources.

## Conflict of Interest

The authors declare that the research was conducted in the absence of any commercial or financial relationships that could be construed as a potential conflict of interest.

## Publisher’s Note

All claims expressed in this article are solely those of the authors and do not necessarily represent those of their affiliated organizations, or those of the publisher, the editors and the reviewers. Any product that may be evaluated in this article, or claim that may be made by its manufacturer, is not guaranteed or endorsed by the publisher.
