# Supplementary material for: A Malaria Parasite Cross Reveals Genetic Determinants of Plasmodium falciparum Growth in Different Culture Media
Source: Front Cell Infect Microbiol. 2022 May 30;12:878496. doi: 10.3389/fcimb.2022.878496 (PMC9197316; doi:10.3389/fcimb.2022.878496)
Supplement: Supplementary file 1 [file DataSheet_1.docx]

**Supplementary Figure 1**


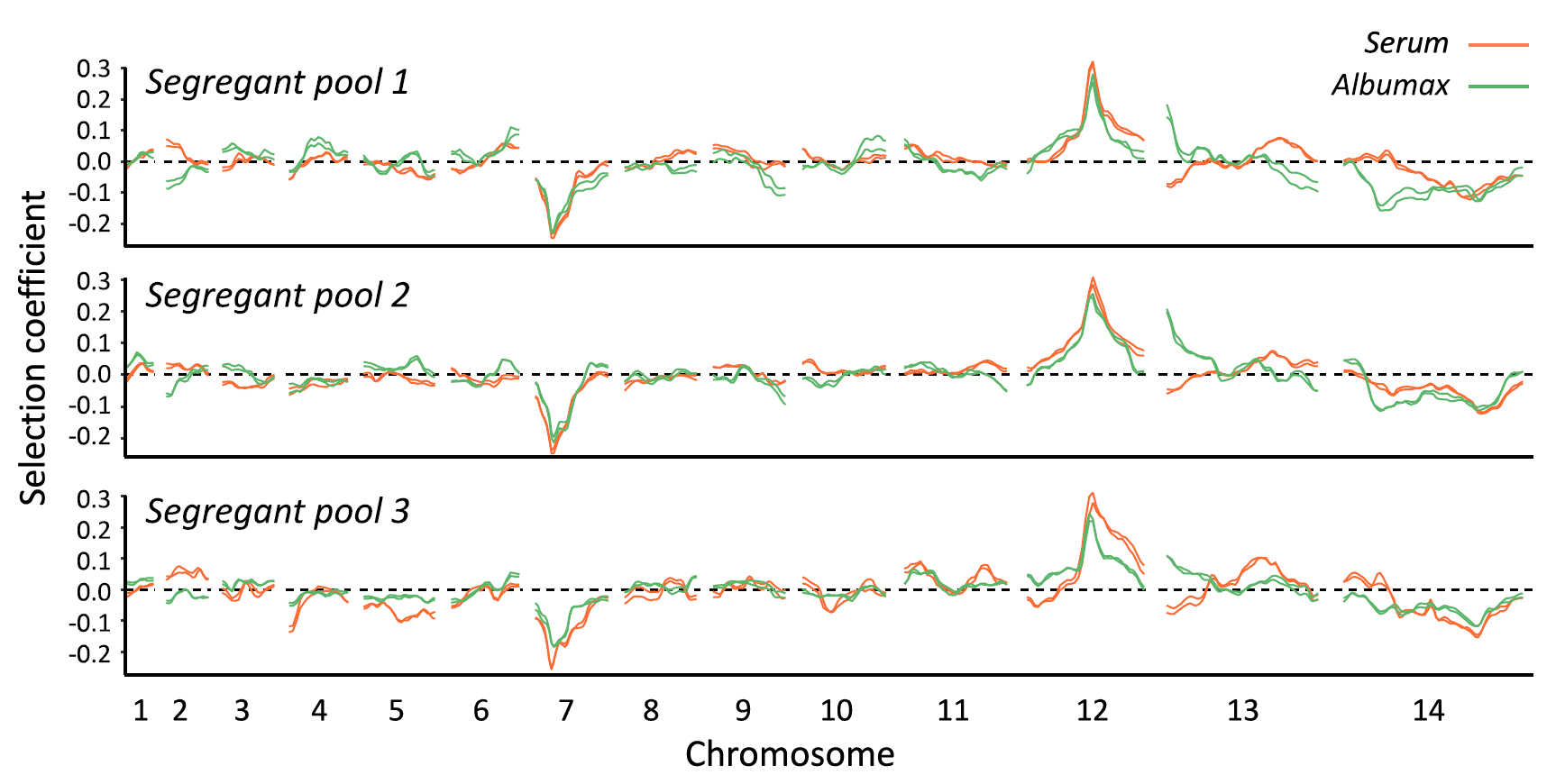


**Supplementary Figure 1.** **Selection coefficients (*s*) across the genome.** Estimation of *s* was based on the changes of allele frequency from day1 to day 30 of cultures. Positive values of *s* indicate a disadvantage for alleles inherited from NHP4026. Orange and green lines indicate cultures by serum and AlbuMAX.

**Supplementary Figure 2**


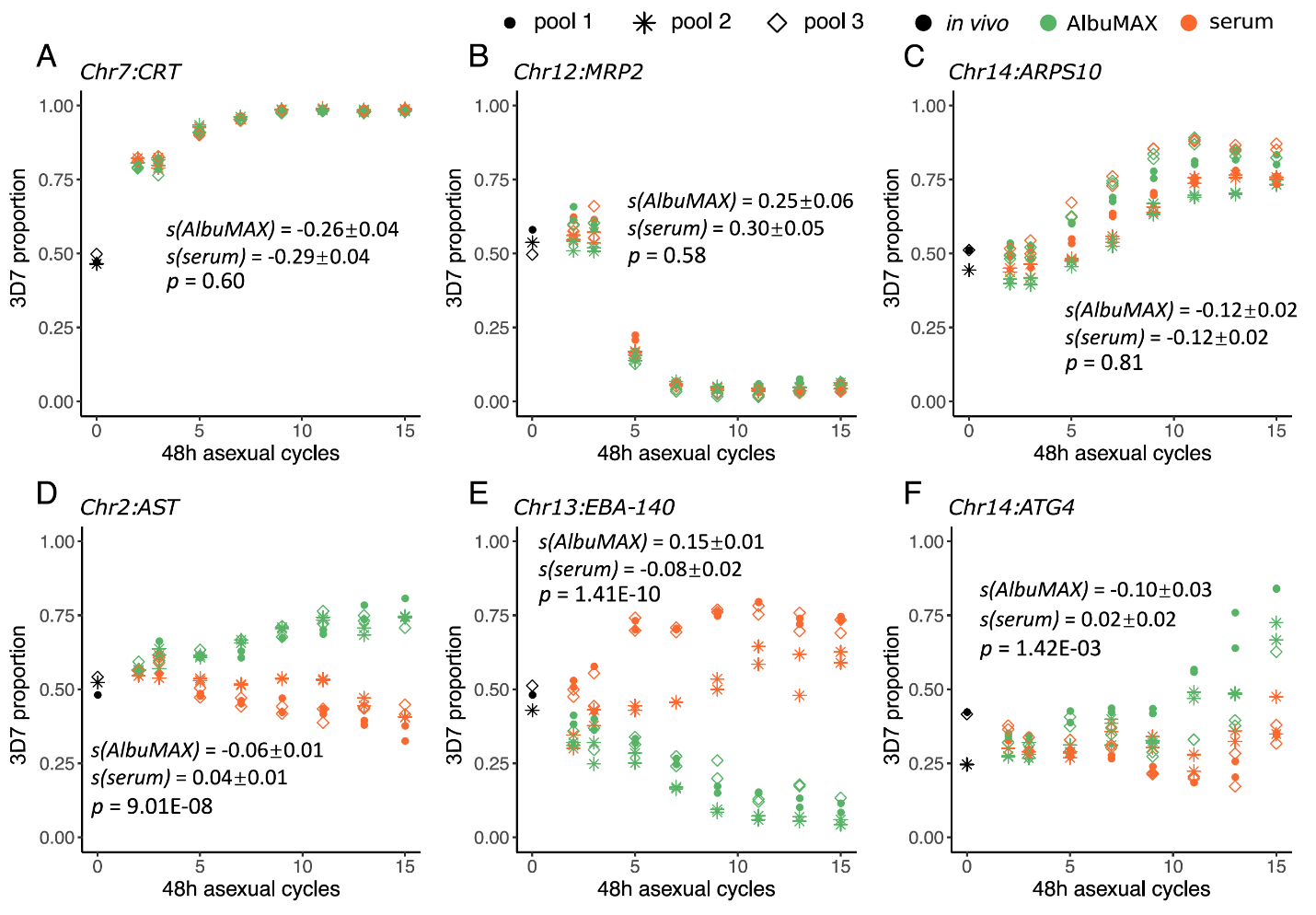


**Supplementary Fig. 2. Estimation of selection coefficients from the changes in allele frequencies in candidate gene regions**. **(A-F)**, 3D7 allele frequency for gene regions of *CRT*, *MRP2*, *ARPS10*, *AST*, *EBA-10* and *ATG4*, separately. Selection coefficients (*s*) were calculated as the slope of the linear model between the natural log of the allele ratio [freq (3D7)/freq (NHP4026)] against time. Positive values of *s* indicate a disadvantage for alleles inherited from NHP4026.

**Supplementary Figure 3**

**
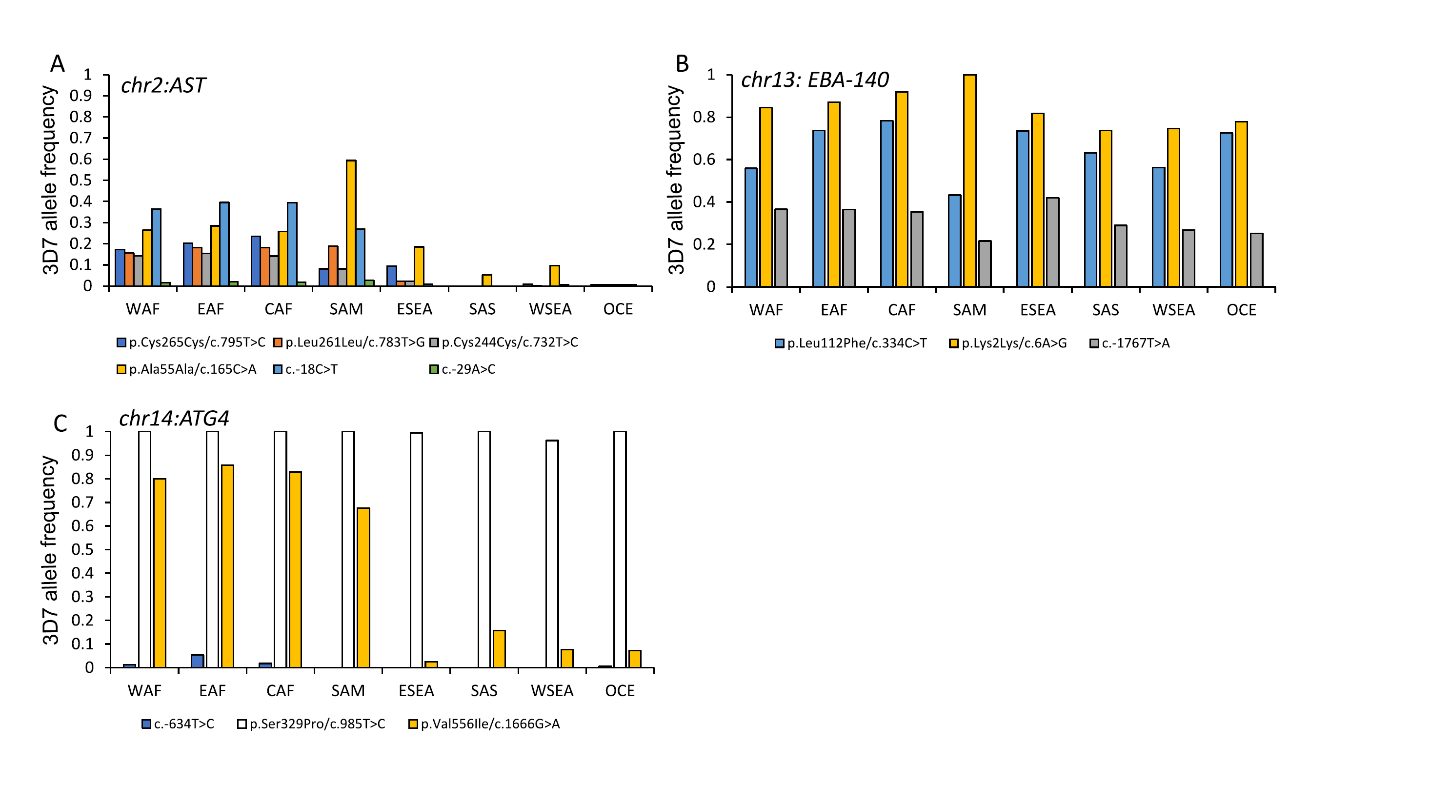
**

**Supplementary Figure 3. 3D7 allele frequency at candidate gene regions in world-wide malaria parasite populations.** (A-C), single nucleotide polymorphisms for gene *AST*, *EBA-10* and *ATG4*, separately. WAF: west Africa, EAF: east Africa, CAF: central Africa, SAM: south America, ESEA: east Southeast (SE) Asia, SAS: south Asia, WSEA: west SE Asia, OCE: Pacific Ocean. The allele frequency analysis was performed using genomic database for *Plasmodium falciparum* (MalariaGEN, <https://www.malariagen.net/>).
